# Ascending Aortic Calcification as a Potential Predictor for Low Bone Mineral Density: A Pilot Study

**DOI:** 10.1155/2021/5526359

**Published:** 2021-05-26

**Authors:** Hirofumi Bekki, Takeshi Arizono, Yuki Suzuki, Akihiko Inokuchi, Takahiro Hamada, Ryuta Imamura, Ryunosuke Oyama, Yuki Hyodo, Eiji Kinoshita, Takumi Kita

**Affiliations:** Department of Orthopaedic Surgery, Kyushu Central Hospital of the Mutual Aid Association of Public School Teachers, Fukuoka, Japan

## Abstract

**Background:**

Identifying the factors related to low bone mineral density (BMD) can have significant implications for preventing hip fractures. The correlation between ascending aortic calcification and BMD has never been reported. Therefore, the purpose of the current study is to confirm the hypothesis that ascending aortic calcification can be used as a predictive factor for low BMD and to find a radiographic sign to show it.

**Method:**

Plain film and computed tomography (CT) images of the thorax were obtained from 91 patients with hip fractures. Using the images, the calcification line of the ascending aorta adjacent to the aortic arch was evaluated. A prominent calcification line confirmed by both plain film and CT was classified as +2. A line which was ambiguous on plain film but confirmed by CT was classified as +1. Cases with no calcification were categorized as 0 (control). We compared the classified score with the BMD and calculated the kappa coefficient to measure intraobserver reliabilities for this radiographic finding.

**Results:**

Twenty-eight patients showed a +2 line, twenty-four patients showed a +1 line, and thirty-nine patients showed 0 lines. The median BMD of each group was 0.37 for the +2 line, 0.45 for the +1 line, and 0.51 for the 0 line. The BMD for the +2 group was significantly lower than the others. The kappa coefficient was approximately 0.6 (*p* < 0.01).

**Conclusion:**

The imaging finding of calcification of the ascending aorta might be considered as a potential surrogate marker of low BMD. In such subjects, BMD might be ordered for the confirmation of diagnosis of osteoporosis. *Mini-Abstract*. The Aortic Arch Tail Sign, a calcification line on the ascending aorta, was relevant to low BMD in the current study. BMD can be ordered for the confirmation of diagnosis of osteoporosis in a subject incidentally found to have ascending aorta calcification on X-ray or CT.

## 1. Introduction

Hip fractures are a severe health problem in patients of advanced age because they can cause a significant decline in mobility and can reduce life expectancy [1]. Japan has become the world's oldest country, with 27.7% of its population being older than 65 years in 2018 [2]. Among clinical risk factors, the bone mineral density (BMD) at the femoral neck is the most robust predictive value for the risk of various fractures [3]. According to research, identifying the factors related to low BMD will have significant implications for the prevention of femoral fractures.

Since the COVID-19 pandemic began in 2020, orthopedic surgeons have been requesting more thoracic CT scans than ever before, particularly for patients requiring emergency surgery.

Radiologic interpretation has shown that many patients with hip fractures also show aortic calcification from the ascending aorta to the aortic arch.

Retrospectively, we also found that aortic calcification was detectable in plain film images. To the best of our knowledge, no studies have examined the relationship between ascending aortic calcification and low BMD in patients with hip fractures. The purpose of this study is to confirm the hypothesis that ascending aortic calcification can be used as a predictive factor for low BMD in the femoral neck among osteoporotic patients.

## 2. Materials and Methods

### 2.1. Patients

We retrospectively reviewed 91 patients with low-trauma hip fractures (42 with trochanteric fractures and 49 with femoral neck fractures) who were all treated at the same medical institution between April and December 2020. All patients received plain film and CT imaging of the thorax in the supine position to avoid COVID-19 infection. BMD (g/cm^2^) in the nonfractured femoral neck was measured by using the Horizon DXA System Bone Densitometer (Hologic Inc., MA). This was because the femoral neck is a consistently significant predictor of hip fractures, and the discriminant power was better than that measured at the lumbar spine [4].

The patient group comprised 74 women and 17 men with a median age of 88 (ranging from 65 to 105 years). The median body mass index (BMI) was 19.8 kg/m^2^ (range, 12.5–30 kg/m^2^).

In accordance with the American Society of Anesthesiologists Physical Status (ASA-PS) scale, 50 cases were classified as grade II and 41 cases were classified as grade III. Chronic diseases, including diabetes mellitus (DM) and hypertension (HT), were checked for because vascular disease is strongly correlated with hip fractures [5, 6]. Diabetes was defined as a patient having a fasting glucose level ≥126 mg/dl or taking hypoglycemic medication. HT was defined as a patient having a systolic blood pressure ≥140 mmHg and a diastolic blood pressure ≥90 mmHg. Thirteen patients had DM, while fifty-eight had HT. Seventy-nine patients received echocardiography to check for aortic stenosis (AS) and aortic regurgitation (AR). This retrospective study was approved by the Kyushu Central Hospital review board (21-1).

### 2.2. Radiographic Evaluation

**Figure 1:**
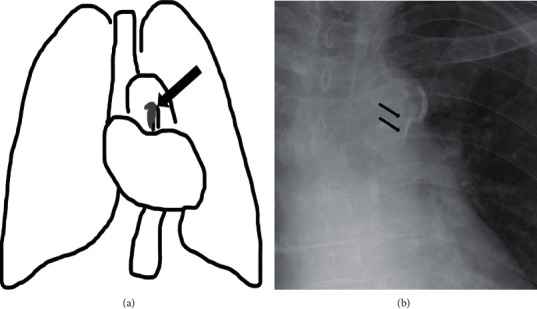
The scheme and representative X-ray images showing the Aortic Arch Tail Sign. The arrows show the calcification line of the ascending aorta adjacent to the aortic arch.

The calcification line of the ascending aorta adjacent to the aortic arch was evaluated from the images. We named this line the “Aortic Arch Tail Sign,” and it is shown in Figure 1. A prominent calcification line confirmed by plain film and CT was classified as +2. A line that was ambiguous in plain film but was validated by CT was classified as +1. Cases with no calcification on the images were classified as 0 (control).

**Figure 2:**
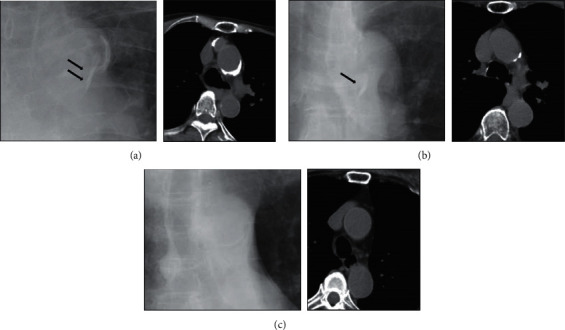
The classification of the Aortic Arch Tail Sign. The prominent calcification line confirmed by plain film and CT imaging was classified as +2. A line which was ambiguous with plain film imaging but was confirmed by CT was classified as +1. Images classified as 0 showed no calcification. (a) +2. (b) +1. (c) 0.

The representative images for each classified score are described in Figure 2. We compared the intensity of the calcification line with the patient data (age, gender difference, BMI, ASA-PS, and past history) and investigated any correlations between the calcification and these parameters.

### 2.3. The Measure of the Rater Agreement

The kappa coefficient was calculated to investigate the intraobserver reliabilities. The kappa coefficient, also known as Cohen's coefficient of agreement, is a widely used index for assessing agreement between raters [7]. Twenty-four out of the ninety-one cases were randomly selected, and two of the co-authors interpreted the plain film images. First, the 24 cases were categorized into groups with or without the Aortic Arch Tail Sign. One week later, they interpreted the same images again and classified all cases into the 0, +1, and +2 groups as described above. The kappa coefficients of the two scenarios were then calculated. We referred to an earlier paper for the interpretation of kappa [8], with a 0.41–0.6 range meaning moderate agreement and a 0.61–0.8 range meaning substantial agreement.

### 2.4. Application of the Aortic Arch Tail Sign to Patients without Hip Fractures

We retrospectively checked plain films among 35 patients without hip fracture over 70 years of age. The patient group composed of 30 women and 5 men with a median age of 80 (ranging from 70 to 89 years). We evaluated whether the presence of the Aortic Arch Tail Sign was relevant to BMD.

### 2.5. Statistical Analysis

All data were expressed as median and within the 25%–75% interquartile range (IQR). After testing for normality using the Shapiro–Wilk test, differences between the two groups were evaluated using Pearson's chi-square test and Student's *t*-test. Nonnormally distributed variables were evaluated using the independent Wilcoxon signed-rank test, while the Kruskal–Wallis and Steel–Dwass tests were used to determine differences in BMD in the +2, +1, and 0 (control) groups. A *p* value of <0.05 was considered statistically significant. The data analysis was conducted using the JMP statistical software package (ver. 15; SAS Institute, Cary, NC).

## 3. Results

**Figure 3:**
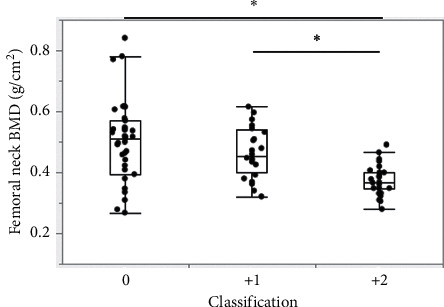
Comparison of BMD according to the Aortic Arch Tail Sign Classification. BMD in the +2-line group was significantly lower than that in the 0- and +1-line groups (^*∗*^*p* < 0.01).

The demographic data are summarized in Table 1. Appropriately 60% cases showed calcification in the ascending aorta. Among the 91 patients, 28 showed a +2 line, 24 showed a +1 line, and 39 showed 0 lines. We also evaluated the presence of calcification in the descending aorta by the CT images for reference, and the positive ratio was 74.7%. There was a significant difference in these positive ratios (*p* < 0.05). The BMI in the +2-line group was significantly lower than that of the 0-line group (18.7 vs. 21.1; *p* <0.01). There were no significant differences in any of the other clinical parameters between the two groups, including gender, age, ASA-PS, type of fracture, medical history, and the electrocardiogram findings. The comparison of BMD according to the Aortic Arch Tail Sign classification is shown in Figure 3. The BMD was 0.37 (0.35–0.4) for the +2-line group, 0.45 (0.4–0.54) for the +1-line group, and 0.51 (0.39–0.57) for the 0-line group. The BMD in the +2-line group was significantly lower than that in the 0- and +1-line groups (*p* < 0.01).

For a measure of rater agreement, the kappa coefficient regarding the presence of the Aortic Arch Tail Sign was 0.61 (95% CI, 0.26–0.95) (*p* < 0.01), and the kappa coefficient for the three classified groups was 0.56 (95% CI, 0.27–0.85) (*p* < 0.01).

For patients without hip fractures, 19 out of 35 (54.2%) were positive for Aortic Arch Tail Sign (median age: 82, 10 women and one man). The mean age and gender difference were similar to those of the group negative for Aortic Arch Tail Sign (median age: 78, 12 women and four men). BMD in the positive group was lower than that in the negative group (0.44 vs. 0.58, *p* < 0.001), but higher than that in the +2-line group with hip fractures (0.44 vs. 0.37, *p* < 0.001).

## 4. Discussion

In the current study, we confirmed the hypothesis that ascending aorta calcification is linked to low BMD in patients with hip fractures. We named the radiographic calcified line in the ascending aorta adjacent to the aortic arch the “Aortic Arch Tail Sign.” Having a prominent Aortic Arch Tail Sign showed a strong correlation with low BMD. Our results suggested that the calcification line of the ascending aorta can be used as a predictive factor for low BMD in the femoral neck among osteoporotic patients.

A relationship between vascular disease and osteoporosis has been reported previously. Vascular calcification is linked to osteoporosis and low BMD in older people or type 2 diabetes patients [9, 10]. Older women with more marked abdominal aortic calcification (AAC) are at a higher risk of fracture [11]. From the point of basic science, arterial calcification processes share some pathways in common with bone physiology, particularly osteoporosis [12]. Iba et al. checked bone-specific alkaline phosphatase, a maker for osteoclast differentiation, and the serum level in osteoporosis patients with abdominal aortic calcification had a higher value than in those without the calcification [13]. Rhee et al. investigated the relationship between Receptor Activator of Nuclear Factor-kappaB Ligand (RANKL) gene polymorphism and aortic calcification [14]. Conversely, however, Aoyagi et al. reported that there was little evidence to support a direct relationship between osteoporosis (low BMD) and aortic calcification by the statistical analysis [15]. In the current study, the Aortic Arch Tail Sign was relevant to low BMD regardless of the presence of hip fractures. However, Aortic Arch Tail Sign in patients without hip fractures did not make a big impact compared to patients with hip fractures. This outcome may be because hip fracture would be strongly correlated with not only low BMD but also the low relative muscle mass [16]. A recent study showed the outcome that severe abdominal aortic calcification was significantly related with hip fracture, but not with vertebral or nonvertebral fractures [17]. For doctors as well as orthopedic surgeons, early detection of aortic calcification could prevent hip fractures before measuring BMD.

As mentioned earlier, since the COVID-19 pandemic began in 2020, orthopedic surgeons have been requesting more thoracic CT scans than ever before, particularly for patients requiring emergency admission or emergency surgery. We retrospectively reviewed CT images of 91 patients, and approximately 60% of them showed aortic calcification from the ascending aorta to the aortic arch. Twenty-eight of the ninety-one patients showed prominent aortic calcification that was easily detectable by plain film imaging. We postulate that this ascending aorta calcification can be used as a predictive factor for low BMD and our named Aortic Arch Tail Sign is significantly correlated with a low BMD. Furthermore, our results showed statistically significant intraobserver reliabilities for the Aortic Arch Tail Sign, which suggested that physicians, as well as orthopedic surgeons, may find this new finding to be a useful predictive tool for low severe osteoporosis.

DXA scan is the gold standard tool for the osteoporosis screening and for the decision regarding antiosteoporotic therapy. In addition, for radiation dose, dosimetry for one examination is around 0.5 mSv, which is equal to that of chest X-ray and much less than 25 mSv of one CT scan. From these points, the utility of chest X-ray remains to be unclear. However, the diffusion rate of medical examination for osteoporosis in 2015 was approximately 5% in Japan. Considering the versality and cost effectiveness of chest X-ray, our Aortic Arch Tail Sign may be used as a “very early screening tool” for osteoporosis before DXA examination.

In our study, the Aortic Arch Tail Sign was also correlated with low BMI. This outcome corresponded with the findings of a previous paper, which implied that low BMI may be associated with increased aortic calcification through calcium mobilization from the bone [18]. However, the other clinical parameters were independent of the Aortic Arch Tail sign. Allison et al. reported that calcifications in all vascular beds increased with age [19], while other studies showed that aortic calcification was more prevalent in women than men [6, 20]. Conversely, Takasu et al. demonstrated that descending thoracic aortic calcification was a predictor of coronary artery calcification but had no relationship with cardiovascular risk factors [20]. More studies might be necessary to find new clinical parameters relevant for the Aortic Arch Tail Sign.

Our study had a limitation. Since aortic calcification is common in the elderly age group, the association between the low BMD and aortic calcification could be a chance finding. However, comparing descending aorta, our data showed the positive ratio for calcified plaque was lower in the ascending aorta. The continuous movement of heartbeat may prevent the plaque formation, and it would lead to the lower frequency to find the calcified change as the descending aorta or abdominal aorta. A previous paper has shown that the etiology of atherosclerotic plaques may differ among aortic tissues [21]. Considering the limited number of previous studies regarding the ascending aorta, the current study is still meaningful despite the small number of patients.

## 5. Conclusions

The Aortic Arch Tail Sign, a calcification line of the ascending aorta adjacent to the aortic arch seen on X-ray and/or CT scan images, was found to be associated with low BMD. Hence, BMD might be ordered for the confirmation of the diagnosis of osteoporosis in subjects incidentally found to have ascending aorta calcification on X-ray or CT.

## Figures and Tables

**Table 1:** Clinical parameters by each classification score.

	+2 (*n* = 28)	+1 (*n* = 24)	0 (*n* = 39)	*p* value^*∗*^
Gender: male/female	8/20	4/20	5/34	0.11
Age (years)	87 (82–92)	88.5 (82.5–93)	88 (80–92)	0.72
BMI	18.7 (17.3–20.5)	20.3 (17.8–21.9)	21.1 (18.6–23.6)	**0.01**

ASA-PS				0.22
2	13	13	24	
3	15	11	15	

Type of fracture				0.07
Neck	11	14	24	
Trochanteric	17	10	15	
DM +/−	6/22	2/22	5/34	0.35
HT +/−	21/7	14/10	23/16	0.17

Electrocardiogram	(*n* = 23)	(*n* = 26)	(*n* = 30)	
EF	65.5 (62–69)	65 (61.2–67)	63 (60–65)	0.07
AS +/−	4/19	6/14	2/28	0.22
AR +/−	17/6	17/3	22/8	0.96

AR: aortic regurgitation, AS: aortic stenosis, ASA-PS: American Society of Anesthesiologists Physical Status, BMI: body mass index, DM: diabetes mellitus, EF: ejection fraction, HT: hypertension, *n*: number of patients. ^*∗*^+2 group vs. 0 group. Pearson's chi-square and Wilcoxon signed-rank test were used.
